# Disbalancing Envelope Stress Responses as a Strategy for Sensitization of *Escherichia coli* to Antimicrobial Agents

**DOI:** 10.3389/fmicb.2021.653479

**Published:** 2021-04-07

**Authors:** Esther Recacha, Valeria Fox, Sara Díaz-Díaz, Ana García-Duque, Fernando Docobo-Pérez, Álvaro Pascual, José Manuel Rodríguez-Martínez

**Affiliations:** ^1^Unidad Clínica de Enfermedades Infecciosas, Microbiología y Medicina Preventiva, Hospital Universitario Virgen Macarena, Seville, Spain; ^2^Red Española de Investigación en Patología Infecciosa (REIPI), Instituto de Salud Carlos III, Madrid, Spain; ^3^Instituto de Biomedicina de Sevilla (IBiS), Hospital Universitario Virgen del Rocío/CSIC/Universidad de Sevilla, Seville, Spain; ^4^Laboratory of Molecular Microbiology and Biotechnology, Department of Medical Biotechnologies, University of Siena, Siena, Italy; ^5^Departamento de Microbiología, Universidad de Sevilla, Seville, Spain

**Keywords:** envelope stress responses, bacterial sensitization, beta-lactams, antimicrobial resistance, Gram-negative bacteria

## Abstract

Disbalancing envelope stress responses was investigated as a strategy for sensitization of *Escherichia coli* to antimicrobial agents. Seventeen isogenic strains were selected from the KEIO collection with deletions in genes corresponding to the σ^E^, Cpx, Rcs, Bae, and Psp responses. Antimicrobial activity against 20 drugs with different targets was evaluated by disk diffusion and gradient strip tests. Growth curves and time-kill curves were also determined for selected mutant-antimicrobial combinations. An increase in susceptibility to ampicillin, ceftazidime, cefepime, aztreonam, ertapenem, and fosfomycin was detected. Growth curves for Psp response mutants showed a decrease in optical density (OD) using sub-MIC concentrations of ceftazidime and aztreonam (Δ*pspA* and Δ*pspB* mutants), cefepime (Δ*pspB* and Δ*pspC* mutants) and ertapenem (Δ*pspB* mutant). Time-kill curves were also performed using 1xMIC concentrations of these antimicrobials. For ceftazidime, 2.9 log_10_ (Δ*pspA* mutant) and 0.9 log_10_ (Δ*pspB* mutant) decreases were observed at 24 and 8 h, respectively. For aztreonam, a decrease of 3.1 log_10_ (Δ*pspA* mutant) and 4 log10_10_ (Δ*pspB* mutant) was shown after 4–6 h. For cefepime, 4.2 log_10_ (Δ*pspB* mutant) and 2.6 log_10_ (Δ*pspC* mutant) decreases were observed at 8 and 4 h, respectively. For ertapenem, a decrease of up to 6 log_10_ (Δ*pspB* mutant) was observed at 24 h. A deficient Psp envelope stress response increased *E. coli* susceptibility to beta-lactam agents such as cefepime, ceftazidime, aztreonam and ertapenem. Its role in repairing extensive inner membrane disruptions makes this pathway essential to bacterial survival, so that disbalancing the Psp response could be an appropriate target for sensitization strategies.

## Introduction

Since antimicrobial resistance is increasing worldwide, new targets (Dickey et al., 2017; Recacha et al., 2017; Cattoir and Felden, 2019) need to be sought, either to find new antimicrobial families or to increase the susceptibility of bacterial populations (Laxminarayan et al., 2013). Envelope stress responses are important pathways for bacterial survival in the presence of stressors, including antimicrobials (Guest and Raivio, 2016; Hersch et al., 2020), and their alteration could be proposed as a strategy for weakening bacteria. The Gram-negative envelope is composed of inner membrane (IM), periplasm, containing a thin peptidoglycan (PG) layer, and outer membrane (OM). This envelope provides Gram-negative bacteria with protection against external environmental agents, including antibiotics (Silhavy et al., 2010). The σ^E^, Cpx, Rcs, Bae and Psp systems are the main envelope stress response pathways in Gram-negative bacteria for restoring homeostasis to cells with induced envelope damage and are activated in different ways (Guest and Raivio, 2016; Mitchell and Silhavy, 2019). The σ^E^ response detects perturbations in outer membrane (OM) or lipopolysaccharide (LPS) biogenesis through interactions between either the exposed C-terminus of misfolded outer membrane proteins (OMPs) and the DegS periplasmic protease, or between the anti-anti-s factor RseB and periplasmic LPS molecules, respectively. These both initiate a regulated intramembrane proteolysis cascade ultimately leading to the liberation of σ^E^ from a membrane-bound anti-sigma factor and the upregulation of adaptive factors, including chaperones, proteases, membrane biogenesis proteins, and a set of small RNAs that downregulate OMP production (Ades, 2004; Ruiz and Silhavy, 2005; Valentin-Hansen et al., 2007; Lima et al., 2013; Flores-Kim and Darwin, 2014; Kim, 2015). The Cpx response is regulated by the CpxA sensor kinase and response regulator CpxR. Envelope stresses causing protein misfolding, and adhesion, inactivate the inhibitor CpxP, trigger CpxA-mediated phosphorylation of CpxR, and altered expression of protein foldases and proteases, respiratory complexes, transporters, and cell wall biogenesis enzymes that impact resistance to a number of antibiotics, particularly aminoglycosides (Raivio, 2014). The Rcs response is regulated by a two-component phosphorelay consisting of two inner membrane (IM)-associated sensor kinase molecules, RcsC and RcsD, together with a cytoplasmic response regulator, RcsB. Multiple environmental parameters and conditions leading to a weakened envelope activate RcsC and/or RcsD, which together catalyze the phosphorylation of RcsB, leading to changes in the expression of genes associated with capsule production, motility, virulence, biofilm formation, and other envelope proteins (Majdalani and Gottesman, 2005; Huang et al., 2006). The Rcs pathway has been linked to resistance to a number of microbially and host produced antimicrobials including beta-lactam antibiotics, cationic antimicrobial peptides and bile (Hirakawa et al., 2003; Erickson and Detweiler, 2006; Laubacher and Ades, 2008; Farris et al., 2010; Farizano et al., 2014). The Psp response is activated by changes linked to the aberrant localization of OM secretin complexes and other conditions that disrupt the IM, including the dissipation of the proton motive force. These signals are transduced through changes in interactions between a set of Psp proteins that ultimately lead to the liberation of the PspF transcription factor from the inhibitor PspA and the upregulated production of a limited set of adaptive factors capable of fostering endurance and survival (Flores-Kim and Darwin, 2014, 2016). Finally, Bae response is controlled by the two-component system made up of the sensor kinase BaeS and its cognate partner BaeR. This pathway is activated by antimicrobial compounds made by plants, animals, and microbes, as well as metals, and can stimulate resistance to broad classes of these substances, primarily, it appears, through the regulation of the multidrug RND efflux pumps AcrD and MdtABC, together with the common OM component TolC (Baranova and Nikaido, 2002; Raffa and Raivio, 2002; Cordeiro et al., 2014; Lin et al., 2014).

The aim of this study was to investigate the effect of alteration of the envelope stress response pathways of the σ^*E*^, Cpx, Rcs, Bae, and Psp systems on sensitization to antimicrobial agents targeting the bacterial cell wall, protein, RNA, DNA or folic acid synthesis.

## Materials and Methods

### Bacterial Strains

A set of 17 *E. coli* strains derived from *E. coli* BW25113 belonging to the KEIO collection were used (Baba et al., 2006). Strains were selected with defective envelope stress responses, with deletions in genes for the σ^E^ (*rseA* and *rseB* genes), Cpx (*cpxA*, *cpxR*, *cpxP*, and *nlpE* genes), Rcs (*rcsF*, *rcsA*, *rcsC*, *rcsD*, and *rcsB* genes), Bae (*baeR* and *baeS* genes) and Psp responses (*pspA*, *pspB*, *pspC*, and *pspF* genes) (Supplementary Table 1). Each deletion was verified by PCR (Supplementary Table 2).

### Antimicrobial Susceptibility Testing

Antimicrobial susceptibility was determined by disk diffusion (Oxoid^®^, United Kingdom) and gradient strip tests (Liofilchem^®^, Italy), using CLSI reference methods (Clinical and Laboratory Standards Institute, 2016.). Any mutant-antimicrobial combination with a halo size that differed by more than 3 mm by disk diffusion from the wild-type (*E. coli* BW25113) was selected for the gradient strip test.

The antimicrobials used were: penicillin G, ampicillin, amoxicillin/clavulanic acid, cefoxitin, ceftazidime, cefepime, ertapenem, imipenem, aztreonam, gentamicin, amikacin, tetracycline, chloramphenicol, colistin, rifampicin, nalidixic acid, ciprofloxacin, sulfonamides compound, sulfamethoxazole/trimethoprim, and fosfomycin.

### Growth Curve Assays

Growth curves were performed for mutant-antimicrobial combinations with a decrease of MIC determined by gradient strip tests. Psp mutants (except Δ*pspF*) were tested to betalactams agents listed in Table 1 despite not showing decreases in MIC value. After overnight culture in Mueller-Hinton broth (MHB) at 37°C, bacterial suspensions were diluted to achieve an OD_625nm_ of 0.1 (*ca.* 10^8^ CFU/mL), then diluted 10^–4^-fold in MHB medium containing sublethal concentrations (0.5xMIC and 0.25xMIC relative to wild-type) of each antimicrobial agent. One hundred and fifty microliters of the diluted bacterial culture were then distributed among 96-well transparent flat bottom plates (Greiner Bio-One, Germany). Cultures were incubated at 37°C on an orbital shaker and agitated (2-mm orbital shaking, 450 rpm, 10 s) for 24 h, and measured with an Infinite 200 PRO plate reader (Tecan). Optical density (OD_595_) measurements were obtained every 20 min. At least 4 biological replicates were measured for each condition in at least two independent assays.

**TABLE 1:** Susceptibility test determined by gradient strip tests.

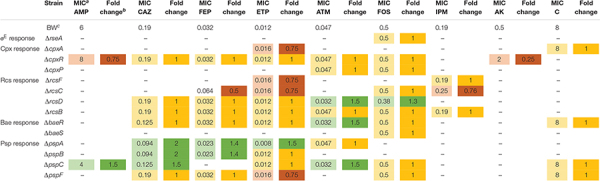

*AMP, ampicillin; CAZ, ceftazidime; FEP, cefepime; ETP, ertapenem; ATM, aztreonam; FOS, fosfomycin; IPM, imipenem; AK, amikacin; C, chloramphenicol.*

*^*a*^MIC (mg/L) of antimicrobial agent by gradient strip test.*

*^*b*^Fold reduction of MIC compared to the MIC of strains (wild-type SOS response).*

*^*c*^Wild-type (E. coli BW25113).*

*Cells with no data correspond to mutant-antimicrobial combinations that were not determined.*

*Green cells- susceptibility increased to antimicrobials; Yellow cells- No changes in susceptibility; Red cells- Resistance increased (relative to wild-type).*

### Time-Kill Curve Assays

To show the effect of alteration of the stress response pathways on bacterial viability, time-kill curve assays were performed with the Δ*pspA*, Δ*pspB* Δ*pspC* mutants. MHB with 1xMIC concentrations of ceftazidime (CAZ), cefepime (FEP), ertapenem (ETP), ampicillin (AMP), and aztreonam (ATM) were used. Antimicrobial concentrations were relative to the MICs for strains harboring unmodified stress responses (wild-type). Growth in drug-free broth was evaluated in parallel as a control. Cultures were incubated at 37°C with shaking at 250 rpm. An initial inoculum of 10^5^ CFU/mL was used in all experiments; bacterial concentrations were determined at 0, 2, 4, 6, 8, and 24 h by colony counting.

### Statistical Analysis

All statistical analyses were performed using Graphpad Prism 6 software^1^. The Student’s *t*-test was used for statistical evaluation when two groups were compared. Differences were considered significant when *P* < 0.05.

## Results

### Sensitization of *E. coli* to Antimicrobials Agents Determined by Disk Diffusion and Gradient Strip Test

Twenty antimicrobials were tested by disk diffusion (Supplementary Table 3) in the initial screening (340 mutant-drug combinations were tested). Psp response was the most sensitized stress pathway with 22.5% of drug-gene deletion combinations affected, followed in descending order, by the Rcs (18%), Bae (17.5%), Cpx (13.7%), and σ^E^ responses (2.5%). To confirm these data, the gradient strip test (Table 1) was used to evaluate the activity of 9 antimicrobials (ampicillin, ceftazidime, cefepime, ertapenem, imipenem, aztreonam, amikacin, chloramphenicol, and fosfomycin) in 14 mutants (Δ*rseA*, Δ*cpxA*, Δ*cpxR*, Δ*cpxP*, Δ*rcsF*, Δ*rcsC*, Δ*rcsD*, Δ*rcsB*, Δ*baeR*, Δ*baeS*, Δ*pspA*, Δ*pspB*, Δ*pspC, and* Δ*pspF*). The mutants that showed antimicrobial sensitization were the following: Δ*rcsD* showed a consistent 1.5- and 1.3-fold decrease in MIC values of aztreonam and fosfomycin, respectively; Δ*baeR*, showed a 1.5-fold decline in the MIC of aztreonam; Δ*pspA* showed 2-, 1.4-, and 1.5-fold decreases in the MICs of ceftazidime (Supplementary Figure 1), cefepime and ertapenem, respectively; Δ*pspB* showed a 2- and 1.4-fold decrease in the MIC of ceftazidime (Supplementary Figure 1) and cefepime, respectively, and finally, Δ*pspC* showed a 1.5-fold decrease in the MICs of ampicillin, ceftazidime and aztreonam.

**FIGURE 1:**
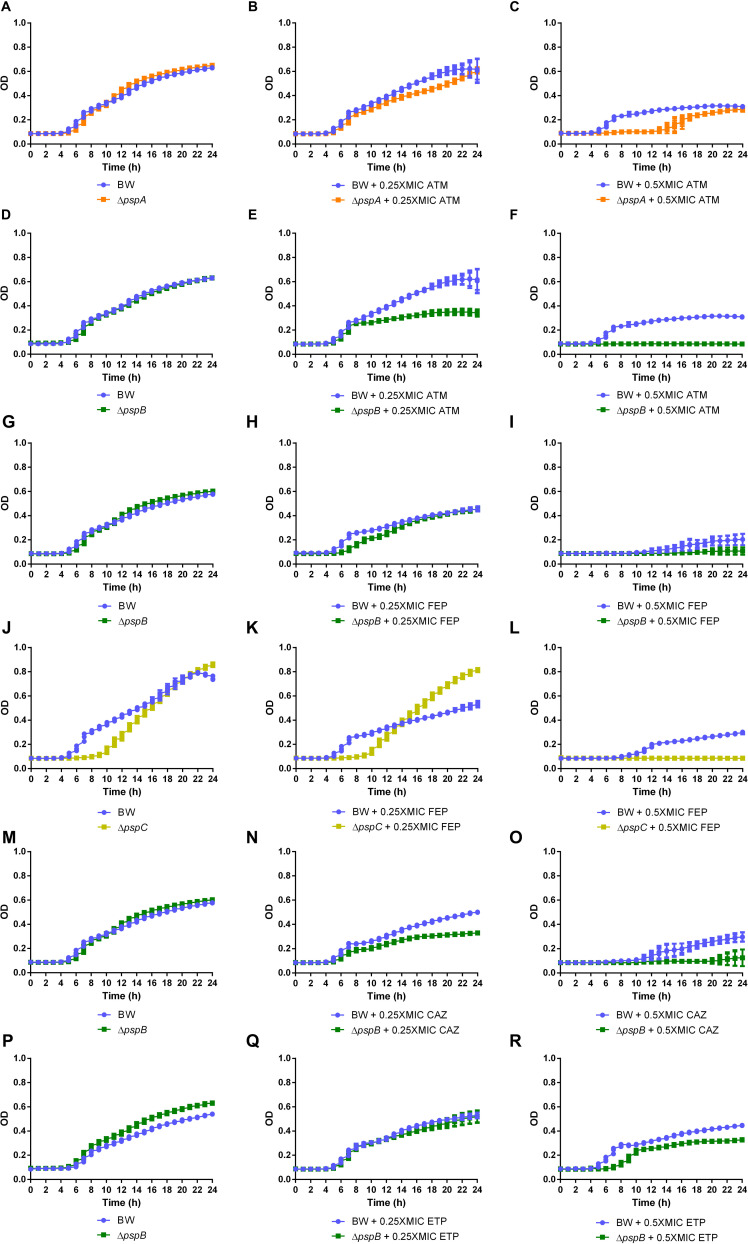
Growth curves in the presence of aztreonam (ATM) for Δ*pspA* **(B,C)** and Δ*pspB* **(E,F)** mutants, cefepime (FEP) for Δ*pspB* **(H,I)** and Δ*pspC* **(K,L)** mutants, ceftazidime (CAZ) for Δ*pspB* **(N,O)** mutant and ertapenem (ETP) for Δ*pspB* **(Q,R)** mutant at concentrations of 0.25xMIC and 0.5xMIC relative to wild-type (BW25113) and their respective controls without antimicrobials **(A,D,G,J,M,P)**.

Each mutant-antimicrobial combination that showed sensitization by gradient strips was tested with growth curves to analyze bacterial growth after short and long incubation periods in the presence of the antimicrobials cited above to confirm the previous results. The generalized sensitization of Psp mutants to beta-lactam agents (Table 1) led to growth curves even although no changes in MIC values were observed. The Δ*pspB* mutant showed clear sensitization with differences for aztreonam, ceftazidime, cefepime and ertapenem relative to wild-type. After 24 h, no growth was observed in the presence of aztreonam at 0.5xMIC (optical density, OD value 0.32) (*p* < 0.01, compared to wild-type, OD value 0.08) (Figures 1D–F), a decrease in OD was observed in the presence of ceftazidime at 0.25xMIC (OD value 0.33) (*p* < 0.0001, compared to wild-type BW25113, OD value 0.5) and at 0.5xMIC (OD value 0.16) (p < 0.05, relative to wild-type, OD value 0.3) (Figures 1M–O), and also in cefepime at 0.5xMIC (OD value 0.11) (*p* < 0.05, relative to wild-type, OD value 0.21) (Figures 1D–F). After 8 h of incubation, a decrease in OD was also observed at 0.25xMIC of cefepime (OD value 0.13) (*p* < 0.0001, compared to wild-type, OD value 0.25) (Figures 1G–I) and at 0.5xMIC of ertapenem (OD value 0.14) (*p* < 0.0001, relative to wild-type, OD value 0.29) (Figures 1P–R). The Δ*pspA* and Δ*pspC* mutants showed no growth at 0.5xMIC of aztreonam (OD value 0.10) (*p* < 0.01, compared to wild-type, OD value 0.27) (Figures 1A–C) until 12 h and no growth was observed at 0.5xMIC of cefepime (OD value 0.08) (*p* < 0.0001, relative to wild-type, OD value 0.30) (Figures 1J–L) at 24 h, respectively. No significant decrease in growth was observed for other mutant-antimicrobial combinations, and a paradoxical effect was observed with aztreonam (Supplementary Figures 2–4).

### The Impact of Psp Response Alteration on Bactericidal Activity of Beta-Lactam Antimicrobials

**FIGURE 2:**
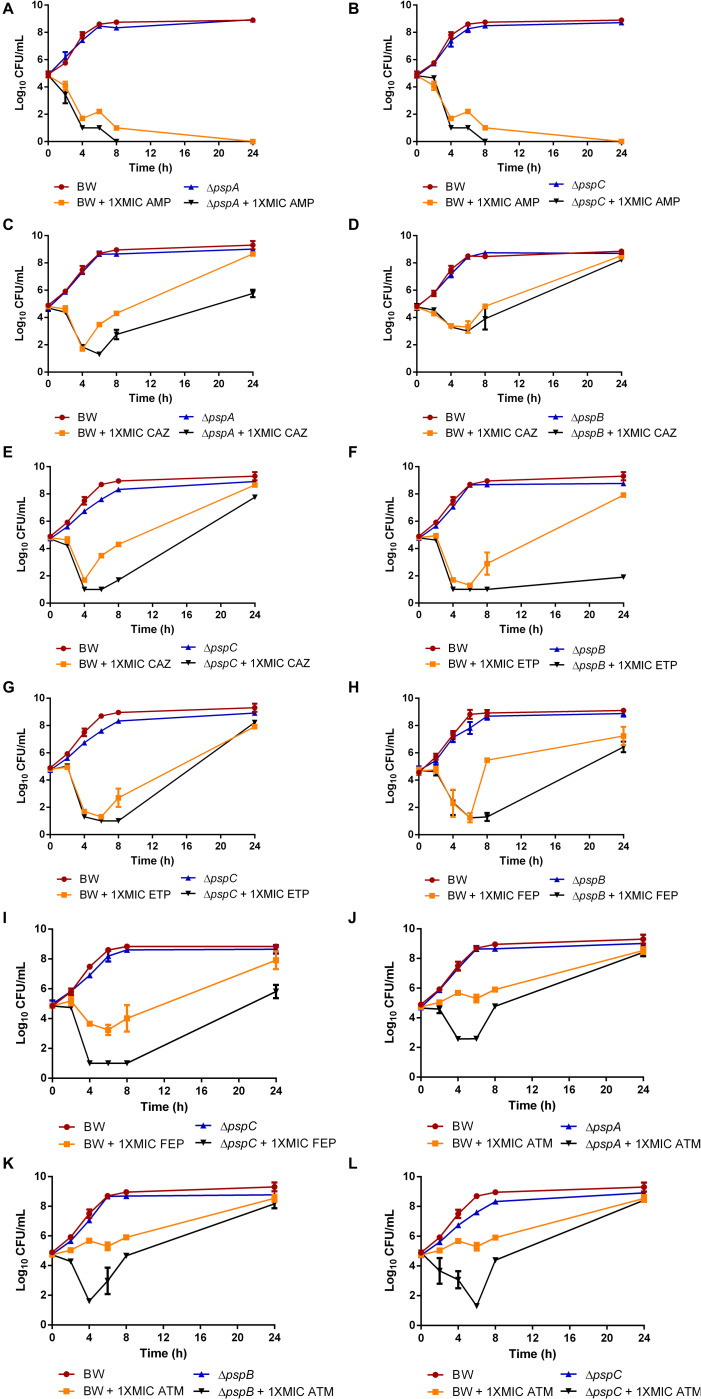
Time-kill curves in the presence of ampicillin (AMP) for Δ*pspA* and Δ*pspC* mutants **(A,B)**, ceftazidime (CAZ) for Δ*pspA*, Δ*pspB*, and Δ*pspC* mutants **(C–E)**, ertapenem (ETP) for Δ*pspB* and Δ*pspC* mutants **(F,G)**, cefepime (FEP) for Δ*pspB* and Δ*pspC* mutants **(H,I)**, aztreonam (ATM) for Δ*pspA*, Δ*pspB*, and Δ*pspC* mutants **(J–L)** at 1xMIC relative to wild-type (BW25113) using low initial inoculum.

Psp mutants (Δ*pspA*, Δ*pspB*, and Δ*pspC*) were the selected mutants due to its significant sensitizing effect to antimicrobials compared to the wild-type and was therefore used for time-kill assays to study cell viability in the presence of 1xMIC concentrations of beta-lactam agents selected. A bactericidal effect was observed for Δ*pspA* and Δ*pspC* mutants in the presence of AMP with drops up to 1 log_10_ (*p* < 0.01) at 8 h (Figures 2A,B). To note, a bacteriostatic effect was observed with the rest of the antimicrobials evaluated. At 1xMIC CAZ and ATM for Δ*pspA*, Δ*pspB*, and Δ*pspC* mutants, reductions of 2.2 log_10_ (*p* < 0.0001), 0.9 log_10_ (*p* = 0.117, ns) and 2.5 log_10_ (*p* < 0.0001), respectively, were observed at 6–8 h for the first agent (Figures 2C–E), maintaining growth delay at 24 h and drops of 3.1 log_10_ (*p* < 0.01), 4 log_10_ (*p* < 0.01) and 3.9 log_10_ (*p* < 0.01), respectively, were observed at 4–6 h for the second drug (Figures 2J–L). At 1xMIC ETP, reductions of 6 log_10_ (*p* < 0.01) for Δ*pspB* mutant and 1.7 log_10_ (*p* < 0.05) for Δ*pspC* mutant were observed at 24 and 8 h, respectively (Figures 2F,G) and drops of 4.2 log_10_ (*p* < 0.0001) for Δ*pspB* mutant and 3 log_10_ (p < 0.01) for Δ*pspC* mutant were found after treatment with FEP at 8 h (Figures 2H,I). No differences in cell viability loss were observed for Δ*pspB* mutant in the presence of ampicillin or for Δ*pspA* mutant in the presence of ertapenem and cefepime (Supplementary Figures 5A–C).

## Discussion

Apart from the search for new drugs, new strategies are also necessary to prevent the emergence of resistance and extend the life of antimicrobial agents. Envelope stress responses are a set of coordinated physiological mechanisms that sense envelope damage or defects and trigger transcriptome alterations to mitigate this stress. In general terms, these pathways are focused on outer membrane stress (σ^E^ response), inner membrane stress (Cpx, and Psp responses), damage through exposure to toxic molecules (Bae response) and alterations in outer membrane permeability, changes in peptidoglycan biosynthesis and defects in lipoprotein trafficking (Rcs response) (Mitchell and Silhavy, 2019). Psp-activated IM disruptions tend to be more severe than those required to activate Cpx, being the first extensive disruptions that result in the loss of proton motive force (van der Laan et al., 2003; Maxson and Darwin, 2004; Becker et al., 2005), which could explain the greater effect on sensitization in strains deficient in this response.

Previous studies have evaluated the effect of mutations on the envelope stress response through deletion of certain genes (Nicoloff et al., 2017) or overactivation responses (McEwen and Silverman, 1980; Cosma et al., 1995; Danese et al., 1995), with sensitization to antimicrobials infrequently used in clinics (rifampicin or bacitracin) (Nicoloff et al., 2017) when RseA was deleted. In the present study, we evaluated a putative strategy consisting of disbalancing of envelope stress responses in the presence of antimicrobials from different families, including cell wall-disturbing agents (penicillins, cephalosporins, carbapenems, aztreonam, colistin, and fosfomycin), protein synthesis inhibitors (aminoglycosides, tetracyclines, and chloramphenicol), RNA synthesis inhibitors (rifampin), DNA synthesis inhibitors (fluoroquinolones) and folic acid synthesis inhibitors (sulfonamides and trimethoprim). It is important to highlight the clinical relevance of our set of selected antimicrobials for the treatment of infections caused by Gram-negative bacteria and the wide spectrum of targets covered, listed above. Gene deletions that affected sensitization to antimicrobials involved the Rcs response (*rcsD*, aztreonam and fosfomycin), the Bae response (*baeR*, aztreonam) and the Psp response (*pspA*, ceftazidime, cefepime and ertapenem; *pspB*, ceftazidime and ertapenem; *pspC*, ampicillin, ceftazidime, aztreonam). Beta-lactams constituted 83% of the antimicrobials to which strains were sensitized by gradient strip test, and the cell wall was the target in 100% of them.

Alteration of the Psp response was the envelope stress pathway with the greatest effect on sensitization in the presence of antimicrobials, as demonstrated by growth curves and time-kill curve assays. Various components are involved in the Psp response, notably PspB (inner membrane protein). Under activating conditions, PspB and PspC interact with PspA (PspF inhibitor), which releases PspF (response regulator) (Yamaguchi et al., 2013), which interacts with RNA polymerase to increase *psp* gene transcription (Jovanovic et al., 1996; Lloyd et al., 2004). Specifically, deletion of the *pspA, pspB and pspC* genes had the greatest impact on cell viability and bacterial growth in the presence of beta-lactams antimicrobials, mainly ampicillin, aztreonam, cefepime, ceftazidime and ertapenem, enhancing the bactericidal effect of this family of agents.

Another important aspect is that the target of these antimicrobials in the cell wall of *E. coli* is primarily PBP3 (ampicillin, cefepime, ceftazdime, aztreonam) and PBP2 (ertapenem) which are involved in cell division whose inhibition lead to filamentation and the formation of spherical cells, respectively (Hayes and Orr, 1983; Bush and Bradford, 2016; Rodvold et al., 2018). Nevertheless, the underlying interaction between these agents and the Psp response proteins is unknown. We could hypothesize that the double damage of the bacterial envelope: inner membrane damage due to *psp* deletion and the beta-lactam antimicrobials effect acting on the PBPs proteins, trigger a sensitization effect reducing bacterial growth a increasing antimicrobial lethality.

In general terms, the effect on antimicrobial sensitization in the tested mutants was moderate but consistent; however, it could be interesting to test other essential genes (*rpoE, degS, rseP, cpxQ, igaA*) involved in envelope stress responses, although these were not available in the KEIO collection.

The emergence of innovative therapeutic strategies, in combination with more conventional approaches, is advancing our understanding of interactions between microbiota, host and pathogenic bacteria. Questions that remain to be answered include how disruption of the envelope stress response could impact not only harmful bacteria, but also healthy ones, causing microbiota impairment and associated disorders, such as *C. difficile* infection (Bäumler and Sperandio, 2016).

In conclusion, a defective Psp envelope stress response increases *E. coli* susceptibility to beta-lactams antimicrobials, and is particularly remarkable with aztreonam, cefepime, ceftazidime and ertapenem. The role of this system in repairing extensive disruptions to the inner membrane makes this pathway essential to bacterial survival. Its use as a potential target for bacterial sensitization deserves in-depth evaluation.

## Data Availability Statement

The raw data supporting the conclusions of this article will be made available by the authors, without undue reservation.

## Author Contributions

VF, SD-D, and AG-D performed the lab assays. ER interpreted the data and wrote the manuscript with input from all co-authors. JR-M and ER designed and guided the execution of all experiments. FD-P, ÁP, and JR-M supervised the project and contributed to the interpretation of the results and valuable discussion. All authors contributed to the article and approved the submitted version.

## Conflict of Interest

The authors declare that the research was conducted in the absence of any commercial or financial relationships that could be construed as a potential conflict of interest.

## Footnotes

1 https://www.graphpad.com
