# Supplementary material for: Disbalancing Envelope Stress Responses as a Strategy for Sensitization of Escherichia coli to Antimicrobial Agents
Source: Front Microbiol. 2021 Apr 7;12:653479. doi: 10.3389/fmicb.2021.653479 (PMC8058218; doi:10.3389/fmicb.2021.653479)
Supplement: Supplementary file 1 [file Data_Sheet_1.docx]

SUPPLEMENTARY MATERIALS

Table S1. Strains and characteristics

| **Strain** | **Genetic description** | **Gene function** | **Envelope stress response pathway** | **Use in this study** | **Reference** |
| --- | --- | --- | --- | --- | --- |
| *E. coli* BW25113 | wild-type *E. coli* | - | - | Susceptibility test, growth curves, killing curves | - |
| ∆*rseA* | *E. coli* BW 25113 Δ*rseA* | σ^E^ inhibitor. Inner membrane protein binds directly to σ^E^,  preventing its association with RNA polymerase | σ^E^ response | Susceptibility test | Keio collection^11^ |
| ∆*rseB* | *E. coli* BW 25113 Δ*rseB* | Inhibits RseA cleavage by DegS (protease) binding to the periplasmic domain of RseA. It stimulates the binding of RseA to σ^E^ | σ^E^ response | Susceptibility test | Keio collection |
| ∆*cpxA* | *E. coli* BW 25113 Δ*cpxA* | Sensor histidine-kinase. Directly responsible for sensing stress. Structural changes in the CpxA sensor domain lead to autophosphorylation of the cytoplasmic histidine-kinase domain. | Cpx response | Susceptibility test | Keio collection |
| ∆*cpxR* | *E. coli* BW 25113 Δ*cpxR* | Response regulator. A phosphate group is transferred from CpxA to receiver domain of CpxR, which activates it for transcriptional regulation. | Cpx response | Susceptibility test | Keio collection |
| ∆*cpxP* | *E. coli* BW 25113 Δ*cpxP* | Inhibition of CpxA activation and  functions via a negative feedback mechanism | Cpx response | Susceptibility test | Keio collection |
| ∆*nlpE* | *E. coli* BW 25113 Δ*nlpE* | Outer membrane lipoprotein which activates the Cpx stress response. It has a direct signalling  role for sensing adhesion to hydrophobic surfaces | Cpx response | Susceptibility test | Keio collection |
| ∆*rcsF* | *E. coli* BW 25113 Δ*rcsF* | Stress sensor protein. This outer membrane lipoprotein senses LPS or peptidoglycan defects. Its aminoterminal residue is located in the outer leaflet and the linker domain is threaded through an OMP, locating the signalling domain of RcsF in the periplasm. | Rcs response | Susceptibility test | Keio collection |
| ∆*rcsA* | *E. coli* BW 25113 Δ*rcsA* | Response regulator. It can act as a heterodimer with RcsB. Unlike RcsB, it is not regulated by phosphorylation, but by Lon protease. | Rcs response | Susceptibility test | Keio collection |
| ∆*rcsC* | *E. coli* BW 25113 Δ*rcsC* | Sensor histidine-kinase. Stress signals activate RcsF and RcsC, which autophosphorylates and phosphorylates RcsD | Rcs response | Susceptibility test | Keio collection |
| ∆*rcsD* | *E. coli* BW 25113 Δ*rcsD* | Phosphotransferase. It phosphorylates RcsB, activating it for transcriptional regulation | Rcs response | Susceptibility test, growth curves | Keio collection |
| ∆*rcsB* | *E. coli* BW 25113 Δ*rcsB* | Response regulator. RcsB alone or as a dimer with other response regulators, such as RcsA, regulates transcription of the Rcs regulon | Rcs response | Susceptibility test | Keio collection |
| *∆baeR* | *E. coli* BW 25113 Δ*baeR* | Response regulator. Activation by BaeS leads to the upregulation of a gene encoding a periplasmic chaperone and efflux pumps as well as genes of unknown function. | Bae response | Susceptibility test, growth curves | Keio collection |
| *∆baeS* | *E. coli* BW 25113 Δ*baeS* | Sensor histidine kinase. The system is activated by exposure to toxic molecules, inducing autophosphorylation of BaeS. BaeS transfers the phosphate group to BaeR (response regulator) to activate it. | Bae response | Susceptibility test | Keio collection |
| *∆pspA* | *E. coli* BW 25113 Δ*pspA* | A PspF inhibitor under non- inducing conditions through physical interaction with it. It also functions as an effector by binding to the inner leaflet of the inner membrane and preventing leakage of protons through the membrane. | Psp response | Susceptibility test, growth curves | Keio collection |
| *∆pspB* | *E. coli* BW 25113 Δ*pspB* | Inner membrane proteins. Under activating conditions, PspB and PspC interact with PspA, which releases PspF. | Psp response | Susceptibility test, growth curves, killing curves | Keio collection |
| *∆pspC* | *E. coli* BW 25113 Δ*pspC* | Inner membrane proteins. Under activating conditions, PspB and PspC interact with PspA, which releases PspF. | Psp response | Susceptibility test, growth curves | Keio collection |
| *∆pspF* | *E. coli* BW 25113 Δ*pspF* | Response regulator.This is an enhancer-binding protein that interacts with RNAP containing σ^N^ (the nitrogen-regulation sigma factor) to increase the transcription of *psp* genes | Psp response | Susceptibility test | Keio collection |

Table S2. Primers used to verify the deletions of the genes analyzed in this study (designed on the sequence of *E. coli* MG1655, https://ecocyc.org/)

| Gene | Primer sequence (5´🡪3´) | Distance to ATG | Primer length | Primer name |
| --- | --- | --- | --- | --- |
| *recA* | TCGTCAGGCTACTGCGTATGCAT | 139 | 23 | Pre-H1-*recA* |
| *rseA* | GTCCCTCCCGGAAGATTTACG | 200 | 21 | Pre-*rseA* |
| *rseB* | CTGGGAGTACCTTCTGAAGCgac | 202 | 23 | Pre-*rseB* |
| *cpxA* | CCTGCTCTATTTGCTGGCACagc | 206 | 23 | Pre-*cpxA* |
| *cpxR* | ATGAACTGACTGCCAGCGTTGagg | 200 | 24 | Pre-*cpxR* |
| *cpxP* | TTAATAGGGAAGTCAGCTCTCG | 200 | 22 | Pre-*cpxP* |
| *nlpE* | GTCAGGAACTGAACCGCGAAGcag | 200 | 24 | Pre-*nlpE* |
| *rcsF* | AATTCGTCCAGGCTTATCAGTC | 199 | 22 | Pre-*rcsF* |
| *rcsA* | AAGCTCACTCACATATCGCAAC | 194 | 22 | Pre-*rcsA* |
| *rcsC* | TTGATTGCGTAAGCGGCGTGgat | 210 | 23 | Pre-*rcsC* |
| *rcsD* | ACATCCGCATAATTTCCAGCaat | 230 | 23 | Pre-*rcsD* |
| *rcsB* | ACCAGTGACTTTGCTGCGTTAG | 198 | 22 | Pre-*rcsB* |
| *baeR* | TTTATCGCACCGAAGGTTCCcgc | 174 | 23 | Pre-*baeR* |
| *baeS* | TTTGGTTCACAGCATGTCAGcgt | 166 | 23 | Pre-*baeS* |
| *pspA* | CGCCACTTGTTAGTGTAATTCG | 156 | 22 | Pre-*pspA* |
| *pspB* | TATTGACCAGATGGAAGCGGAAG | 200 | 23 | Pre-*pspB* |
| *pspC* | TTTACCGATCTGGTTATGGCTGC | 173 | 23 | Pre-*pspC* |
| *pspF* | ATGTTGGCATTCACGATGTCggc | 209 | 23 | Pre*-pspF* |
|  | Primer sequence (3´🡪5´) |  | Primer length | Primer name |
|  | CAGTCATAGCCGAATAGCCT |  | 20 | K1^a^ |

a- kanamycin resistance gene from pKD13 (Datsenko and Wanner, 2000)

Table S*3*. Susceptibility test determined by disk diffusion assays, colour-coded according to the difference in halo size (mm) between mutants and wild-type

|  |  | **σ^E^ response** | | **Cpx response** | | | | **Rcs response** | | | | | **Bae response** | | **Psp response** | | | |
| --- | --- | --- | --- | --- | --- | --- | --- | --- | --- | --- | --- | --- | --- | --- | --- | --- | --- | --- |
|  | BW | ∆*rseA* | ∆*rseB* | ∆*cpxA* | ∆*cpxR* | ∆*cpxP* | ∆*nlpE* | ∆*rcsF* | ∆*rcsA* | ∆*rcsC* | ∆*rcsD* | ∆*rcsB* | ∆*baeR* | ∆*baeS* | ∆*pspA* | ∆*pspB* | ∆*pspC* | ∆*psp*F |
| **P** | 8 | 6 | 8 | 6 | 6 | 7 | 6 | 6 | 6 | 6 | 6 | 6 | 6 | 6 | 11 | 6 | 8 | 7 |
| **AMP** | 9 | 9 | 10 | 6 | 14 | 12 | 12 | 12 | 10 | 8 | 10 | 10 | 11 | 9 | 10 | 9 | 15 | 11 |
| **AMC** | 23 | 23 | 24 | 23 | 26 | 24 | 24 | 24 | 24 | 24 | 25 | 24 | 26 | 23 | 26 | 23 | 25 | 26 |
| **FOX** | 28 | 30 | 30 | 28 | 30 | 30 | 29 | 29 | 28 | 30 | 30 | 29 | 31 | 28 | 30 | 29 | 30 | 30 |
| **CAZ** | 30 | 30 | 32 | 30 | 34 | 32 | 32 | 30 | 30 | 32 | 34 | 34 | 34 | 32 | 34 | 34 | 34 | 34 |
| **FEP** | 33 | 36 | 34 | 36 | 38 | 36 | 36 | 36 | 36 | 38 | 38 | 39 | 38 | 36 | 39 | 38 | 36 | 37 |
| **ETP** | 32 | 34 | 34 | 36 | 37 | 34 | 34 | 36 | 34 | 36 | 36 | 39 | 36 | 34 | 37 | 36 | 37 | 36 |
| **IPM** | 28 | 30 | 30 | 30 | 31 | 30 | 30 | 32 | 30 | 32 | 30 | 32 | 28 | 29 | 30 | 30 | 29 | 30 |
| **ATM** | 33 | 33 | 36 | 33 | 38 | 37 | 34 | 34 | 32 | 36 | 37 | 38 | 38 | 36 | 39 | 36 | 38 | 34 |
| **CN** | 28 | 30 | 30 | 28 | 30 | 28 | 28 | 28 | 25 | 30 | 30 | 30 | 30 | 30 | 30 | 28 | 30 | 28 |
| **AK** | 25 | 27 | 27 | 22 | 29 | 26 | 26 | 26 | 22 | 24 | 25 | 28 | 27 | 27 | 27 | 26 | 27 | 25 |
| **TE** | 24 | 26 | 27 | 26 | 27 | 26 | 25 | 24 | 24 | 22 | 26 | 27 | 27 | 27 | 27 | 26 | 27 | 27 |
| **C** | 25 | 28 | 28 | 30 | 28 | 27 | 27 | 24 | 27 | 28 | 28 | 28 | 30 | 28 | 27 | 28 | 31 | 30 |
| **CT** | 18 | 19 | 20 | 20 | 20 | 18 | 18 | 18 | 18 | 21 | 20 | 21 | 19 | 18 | 20 | 19 | 19 | 19 |
| **RD** | 17 | 18 | 18 | 17 | 17 | 17 | 17 | 12 | 16 | 17 | 17 | 18 | 18 | 18 | 17 | 17 | 17 | 17 |
| **NA** | 23 | 24 | 23 | 25 | 24 | 23 | 23 | 22 | 23 | 22 | 24 | 25 | 24 | 24 | 21 | 24 | 24 | 23 |
| **CIP** | 36 | 36 | 36 | 36 | 34 | 36 | 34 | 32 | 32 | 32 | 36 | 38 | 34 | 34 | 34 | 36 | 36 | 34 |
| **S3** | 33 | 32 | 28 | 29 | 32 | 31 | 32 | 32 | 32 | 30 | 30 | 32 | 31 | 32 | 31 | 33 | 31 | 30 |
| **SXT** | 33 | 34 | 34 | 32 | 32 | 32 | 32 | 32 | 32 | 32 | 34 | 34 | 35 | 32 | 32 | 34 | 34 | 32 |
| **FOS** | 27 | 34 | 28 | 28 | 32 | 34 | 28 | 28 | 27 | 35 | 36 | 35 | 32 | 35 | 29 | 26 | 31 | 32 |

P (penicillin G, 1 µg), AMP (ampicillin, 2 µg), AMC (amoxycillin/ clavulanic acid, 30 µg), FOX (cefoxitin, 30 µg), CAZ (ceftazidime, 30 µg), FEP (cefepime, 30 µg), ETP (ertapenem, 10 µg), IPM (imipenem, 10 µg), ATM (aztreonam, 30 µg), K (kanamycin, 30 µg), CN (gentamicin, 30 µg), AK (amikacin, 30 µg), TE (tetracycline, 30 µg), C (chloramphenicol, 30µg), CT (colistin, 50 µg), RD (rifampicin, 30 µg), NA (nalidixic acid, 30 µg), CIP (ciprofloxacin, 5 µg), S3 (sulfonamides compound, 300 µg), SXT (sulfamethoxazole/ trimethoprim, 25 µg), FOS (fosfomycin, 200 µg)

<= 0 mm – yellow; 1-3 mm- orange; 4-6 mm- blue; > 6 mm - green

Figure S1. MIC of ceftazidime for BW25113 and ∆*pspA* and ∆*pspB* mutants, determined by gradient strip test


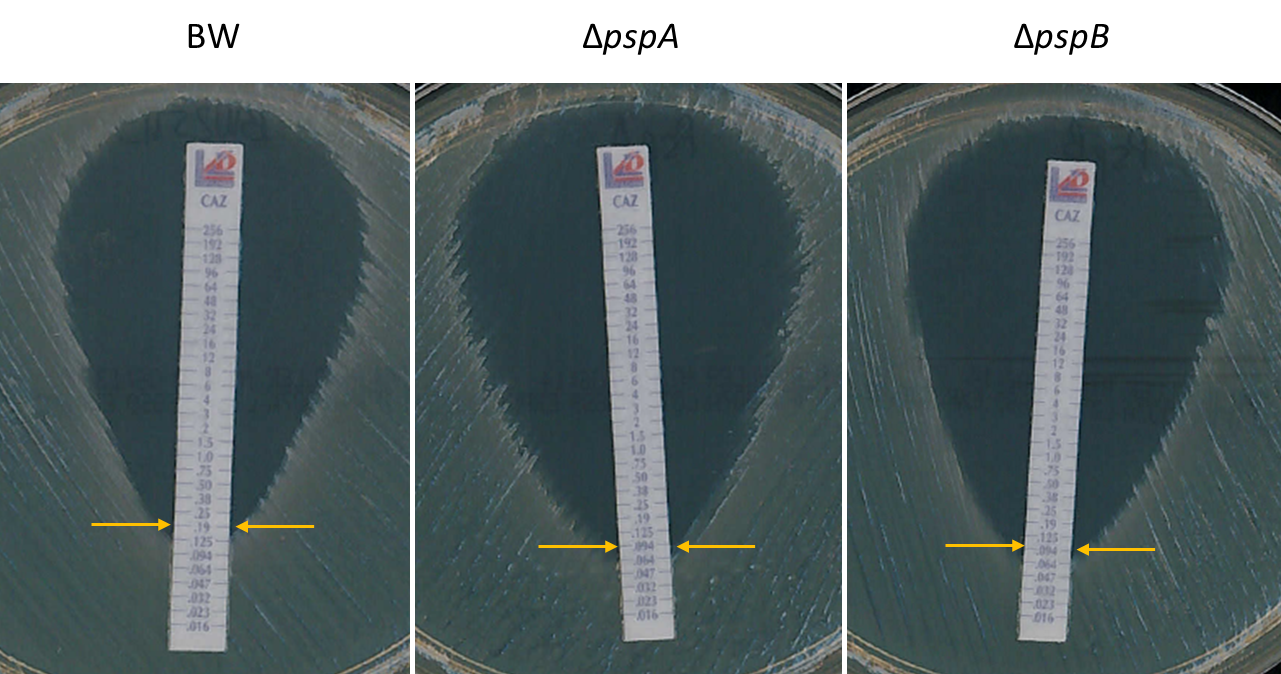


Figure S2. Growth curves for mutants Psp response in the presence of ceftazidime (CAZ), B-C; cefepime (FEP), E-F; ampicillin (AMP), H-I; ertapenem (ETP), K-L and aztreonam (ATM), N-O, at concentrations of 0.25x and 0.5xMIC relative to wild-type and their respective antimicrobial-free controls (A, D, G, J and M)

Figure S3. Growth curves for mutant Rcs response in the presence of aztreonam (ATM), B-C and fosfomycin (FOS), E-F at concentrations of 0.25x and 0.5xMIC relative to wild-type and their respective antimicrobial-free controls (A and D)

Figure S4. Growth curves for mutant Bae response in the presence of aztreonam (ATM), B-C at concentrations of 0.25x and 0.5xMIC relative to wild-type and its respective antimicrobial-free control (A)

Figure S5. Time-kill curves in the presence of ampicillin (AMP) A, ertapenem (ETP) B, and cefepime (FEP) C, at 1xMIC relative to wild-type.
